# The Effect of Oral Function Improvement on Dietary Habits in Older Adults Requiring Support Care

**DOI:** 10.1155/nrp/1531604

**Published:** 2025-06-09

**Authors:** Kazuya Ikenishi, Akio Tada

**Affiliations:** ^1^Faculty of Nursing, Graduate School of Nursing, Hyogo University, Hiraoka 2301, Shinzaike, Kakogawa, Hyogo, Japan; ^2^Faculty of Health Science, Department of Health System Management, Graduate School of Nursing, Hyogo University, Hiraoka 2301, Shinzaike, Kakogawa, Hyogo, Japan

**Keywords:** community-dwelling aged people, diet, masticatory force, oral diadochokinesis, oral function, tongue pressure

## Abstract

**Background:** Aging causes a decline in various functions. Among older adults, deteriorations in oral function might interfere with their diet. However, the insight into whether oral function improvement affects older adults' diet remains unclear.

**Aim:** To investigate whether oral function improvement by an intervention can affect the diet of community-dwelling aged people needing any care.

**Participants and Methods:** This study enrolled 16 community-dwelling older people aged 65 years who were certified to require support care level 1 or 2. We measured the participants' oral function (tongue pressure, oral diadochokinesis, and masticatory force) and used questionnaires to collect data on attributes and diet before and after the intervention, which comprised four oral exercises once a day for 3 months at their own homes. The effect of the intervention was determined using the paired *t*-test and Wilcoxon signed-rank sum test.

**Results:** Tongue pressure (*p* < 0.001), /pa/syllable (*p* = 0.027), /ta/syllable (*p* = 0.046), and masticatory force (*p* = 0.012) significantly improved after the intervention. Conversely, the/ka/syllable (*p* = 0.083) and ingestion frequency (*p* = 0.107 − 0.773) did not change significantly.

**Conclusions:** Oral function training improved the oral function of older adults but could not change their dietary habits. Diet improvement may require dietary counseling together with oral exercise.

## 1. Introduction

The global population of adults aged 65 years and more is approximately 700 million, which is expected to increase to 1.5 billion in 2050 [1]. Hence, the health maintenance of older people is an urgent issue. Aging deteriorates various physical functions and weakens lower-limb muscle strength, leading to a reduced ability in walking and performing activities of daily living (ADLs). Moreover, aging makes living independently difficult among older people. Such people with weakened physical functions caused by aging are frail and are more likely to be hospitalized than those who are not frail [2].

In older adults, oral function decline by aging results from various factors, including suprahyoid and genioglossus muscle weakness, tooth loss, periodontal disease, salivary secretion reduction, hyposmia, and dysgeusia, leading to deterioration in diet, including reduction and change in diet, and consequently, malnutrition. Some cross-sectional studies have reported that masticatory force is significantly related to serum albumin level, weight, and food diversity, supporting the adverse effects of oral function deteriorations on food intake and nutritional status [3–6].

However, in a follow-up study of 4 years, dietary habits did not almost change with no intervention [7] hence, any intervention may be necessary to restore oral function. If restored, diet among older people is expected to improve. Although the significant effects of oral function training have already been widely studied, no study has investigated the effect of oral function training on the diet. Thus, this study aimed to investigate whether oral function improvement by an intervention affects the diet of older adults requiring support care level 1 or 2.

## 2. Participants and Methods

### 2.1. Participants

This study included community-dwelling older people aged 65 years and above who were certified to require support care level 1 or 2 according to Japan's long-term care insurance system (LTCI) guidelines. LTCI is among the social security systems in Japan that provide older people with long-term medical and welfare care service according to their required nursing care level to assist them in the community. Older people requiring support care level 1 or 2 have a slight deterioration in the ability to perform their activities, but with appropriate support as well as frailty, they may perform their ADLs optimally.

District welfare commissioners, managers of welfare facilities, such as day-care centers, and staff of social welfare councils selected our participants. The exclusion criteria were as follows: with difficulty in conversation and writing by him/herself, with tube feeding requirement; with aspiration risk, and with complaints of some pain in the oral cavity during mastication.

### 2.2. Study Design

This prospective cohort, interventional study analyzed the differences between the pre- and postintervention data. The participants were asked to perform an oral exercise once a day for 3 months at their home.

The sample size was estimated according to the results of previous research comparing paired *t*-test results [7]. Before estimating the sample size, we used the following formula of the effect size (Cohen's *d*). The significance level and power were set at 0.05 and 0.80, respectively.

(1) $$\begin{array}{c} \text{Effect size}\frac{\left|M_{A}-M_{B}\right|}{\sqrt{\left(\sigma_{A}^{2}+\sigma_{B}^{2}\right)/2}}, \end{array}$$

where *M*_*A*_ and *M*_*B*_ denote the means after and before the intervention, and *σ*_*A*_ and *σ*_*B*_ indicate their corresponding standard deviations (SDs), respectively.

The sample size (one-sided test) was estimated using G^∗^Power software, which is used in popular academic research. The setting conditions in G^∗^Power (3.1.9.7. Heinrich Heine Universität Düsseldorf, Düsseldorf, Germany) were “Difference between two dependent means (matched pairs)” and “One tail,” α error = 0.05, and power = 0.80.

Additionally, the effect size was 0.998, which is similar to that in the study of Sakayori [8], who reported means and SD and compared pre- and postintervention data with an intervention period of 3 months.

Similar to the tongue pressure study of Van den [9], who conducted the intervention for 8 weeks, the calculated effect size was 2.003.

In this estimation, we did not calculate the mastication force using a color-changeable chewing gum, given that previous studies investigating mastication force did not obtain the means in numerical value by measuring the gum color but by assessing the five visible color levels of the gum as ordinal data. Moreover, all studies without reports on the means in oral function measurements were excluded in the estimation.

According to G^∗^Power sample size determination, a sample size of eight was sufficient to provide the value for oral diadochokinesis (ODK) and five for the tongue pressure analysis. The minimum sample size in a group in this intervention study was five. Consequently, we targeted eight participants in a group.

### 2.3. Data Collection

To clarify the relationship of oral function with diet, muscle strength, and activity, we evaluated the oral function and muscle strength using measurement instruments and questionnaires to collect data on attributes, diet, and activity. We examined demographic factors such as age and sex, and oral functions such as tongue pressure, ODK, and masticatory force.

In particular, tongue pressure was measured using a JMS tongue pressure manometer (TPM-02; JMS Co., Ltd., Hiroshima, Japan), which has a stick with a small balloon on the top. Once this stick was inserted into the oral cavity, the balloon was pushed with the tongue toward the hard palate to measure the tongue pressure. The maximum value displayed on the device monitor served as the tongue pressure.

Assessing ODK evaluates the function of the lips, anterior tongue, and posterior tongue [10], especially the tongue's motor skill. The participant uttered one syllable repeatedly and as quickly as possible for 5 s. The utterances were measured using an oral function measurement device (Kenkou-Kun Handy; Takei Scientific Instruments Co., Ltd., Niigata, Japan). To measure the ODK, we computed the utterance time taken to produce a multisyllable sound (/pataka/) and converted it into the ODK rate by calculating the number of syllables pronounced per second [11].

The masticatory force was measured by chewing a Xylitol chewing gum (Mastication Check Gum; Lotte Co., Ltd., Tokyo, Japan). While several research methods use other foods, the color-changeable chewing gum method is easier and safe, ensuring reliability and validity of mastication [12].

Participants chewed the gum 60 times for approximately 2 min. Chewing the gum will change the gum color from green to red. The depth of red was measured using a color-measuring instrument to convert a numerical value (TES-135A plus; Sato Shoji Corp., Tokyo, Japan).

The red color value is based on *a*^∗^, which means the degree of color between red and green in the Lab color space defined by the International Commission on Illumination. The deeper the red color (more numerical value of red), the stronger the masticatory force.

Diet, specifically regarding the frequency of food ingestion for a week over the last month, was investigated using a Brief Self-Administered Diet History Questionnaire (BDHQ). The original BDHQ consists of 58 food or drink items. However, we only utilized 12 items, excluding drinks and ice cream, among others, because we aimed to evaluate the masticatory force of older people in terms of food hardness. Cuttlefish, octopus, mushroom, and noodles, among others, were also excluded from the survey because these are generally not eaten every day. The 12 food items included “meals” (except for processed meat) and “fish,” “potato,” “green vegetable salad,” “green and yellow vegetable” (broccoli, etc.), “cabbage/Chinese cabbage,” “carrot/pumpkin,” “radish/turnip,” “other root crops,” “seaweed,” “fruit,” and “bread.”

The intervention included four oral exercises to strengthen the muscles involved in oral function. The participants performed the intervention once daily at their home for 3 months. This intervention aimed to achieve the following conditions: 1. lip-closing force reinforcement, 2. mastication muscle reinforcement, 3. tongue motor function improvement, and 4. tongue skill improvement.

The intervention focused on the muscles involved in the oral preparatory stage of swallowing. These muscles include the orbicularis oris, masticatory muscle (masseter, temporal, medial pterygoid, and lateral pterygoid muscles), cheek muscle, suprahyoid muscle, infrahyoid muscle, intrinsic and extrinsic tongue muscles, and sternocleidomastoid muscle, except for the pharyngeal muscles (palatal tensor, palatal elevator, and palatopharyngeal muscles, among others). Aimed at reinforcing muscle strength, this intervention comprised four oral exercises, focusing on the motion involved in the oral preparatory stage. These exercises were used in previous intervention studies, which reported oral function improvement in older adults through these exercises [6, 13–17].

The first section of the intervention aims to enhance the lip-closing force by closing the mouth tightly and puffing the cheek by straining the orbicularis oris and cheek muscle hard. The second section includes pushing the mandibular with the thumbs while also pulling the chin, and pushing the forehead while facing down for 5 s. The third section consists of reinforcing the internal and external tongue muscles. In this section, the following four tongue exercises are performed: extending the tongue forward, retracting it in the direction of the pharynx, expanding it to the bilateral corners of the mouth and the upper and lower lips, and pushing the inside of the bilateral cheek with the tongue. These exercises are expected to improve bolus formation by strengthening the internal and external tongue muscles. In the fourth section, the monosyllables/pa/,/ta/, and/ka/are repeated; these syllables are commonly used to evaluate the movement capacity of the lip and the anterior and posterior buccal regions, respectively. This exercise strengthens the orbicularis oris and cheek muscles as well as the internal and external tongue muscles, similar to the exercises in the first and third sections. This exercise takes approximately 10 min to complete.

The intervention was cancelled if the participant experienced intermittent or continuous oral pain, admitted to any hospital or welfare facility, or had any poor physical condition that made them impossible to perform the exercises.

The investigation period was from October 2020 to October 2022.

### 2.4. Statistical Analysis

Before the comparison analysis, the normality distribution of the collected data was assessed using the Shapiro–Wilk test. To clarify the effect of the intervention on diet change (BDHQ), we implemented paired *t*-test and Wilcoxon signed-rank sum test. A *p* value less than 5% was considered statistically significant. All statistical data were analyzed using IBM SPSS version 27.0 (IBM Japan, Tokyo, Japan). As mentioned, the sample size was calculated using G^∗^Power.

### 2.5. Ethical Considerations

This study obtained approval from the Ethics Committee of Hyogo University (approval no. 20010) and conformed to the guidelines stipulated in the 2013 revision of the Declaration of Helsinki. All participants provided written informed consent.

## 3. Results

Out of 17 individuals consented to participate, one dropped out during the study. Ultimately, 16 older adults completed the 3-month intervention program (6 men, 10 women), with a mean age of 76.6 years (men, 75.0 years; women, 77.4 years). Among them, 11 (6 men, 5 women) and 5 (5 women) were certified as requiring support care levels 1 and 2, respectively (Table 1).

At baseline, the mean values of oral function were as follows: tongue pressure, 29.3 kPa;/pa/syllable, 5.2 times/s;/ta/syllable, 5.2 times/s;/ka/syllable, 5.1 times/s; and masticatory force (color-changeable chewing gum), 27.5.

Among the 12 food items, green vegetable salad, green and yellow vegetables, cabbage/Chinese cabbage, and fruits were eaten four times a week by nearly half of the participants. Approximately ten participants ate fish and potato, carrot/pumpkin, radish/turnip, other root crops, and seaweed one to three times a week. Most of the participants ate meals and bread almost daily at baseline; therefore, these two foods were excluded from the analysis of the change in food ingestion frequency.

After the intervention, the mean values of oral function were as follows: tongue pressure, 34.4 kPa;/pa/syllable, 5.6 times/s;/ta/syllable, 5.7 times/s;/ka/syllable, 5.3 times/s; and masticatory force, 30.1. All of the oral function parameters improved from the baseline values.

Regarding the frequency of food ingestion, fish and green vegetables, salad, carrot, and pumpkin were eaten more than four times per week after the intervention, indicating improvement. The other foods showed no apparent changes as compared with the baseline data.

The Shapiro–Wilk test showed that all oral function data were normally distributed (Table 2).

Tongue pressure (*p* < 0.001), /pa/syllable (*p*=0.004), and/ta/syllable (*p* = 0.018), and masticatory force (*p* = 0.012) significantly improved after the intervention, but the/ka/syllable (*p* = 0.894) did not (Table 3).

The effect sizes in this study showed that the tongue pressure,/pa/syllable, and/ta/syllable were of medium degree (0.547, 0.589, and 0.699, respectively), mastication was of high degree (0.803), and the/ka/syllable was of low degree (0.353).

The frequency of ingesting twelve foods (BDHQ) did not vary significantly (*p* = 0.107 − 0.773) (Table 4).

## 4. Discussion

In this study, oral function training significantly improved participants' tongue pressure, /pa/syllable, and/ta/syllable, and masticatory force, as previously reported [8, 9, 11, 14–21]. The tongue pressure and ODK values improved to almost the same level as that of independent older people described in previous studies [18, 19, 22]. Regarding the masticatory force, comparison with previous studies was impossible; the present study measured the change in the color of chewing gum using an instrument (TES-135A plus), whereas previous studies merely visually judged the change in the color of chewing gum from green to red, which was classified into five grades [18, 20]. The study's masticatory force measurements that were quantized in *a*^∗^ (red color depth) in the Lab color space by the instrument is reportedly more precise than visual judgment in evaluating the small difference of masticatory force [23].

Additionally, given that the participants followed the same frequency in ingesting foods and chewing the gum to minimize variability among measurement methods, the obtained data and the result of improved mastication were confirmed to be highly reliable.

The oral function training utilized in this study exhibited sufficient effects on oral function and is considered to be proper.

Our study demonstrated that the effect of oral function training was marginal in the/ka/syllable. In the studies of Sugiyama [14] and Kito [18], only the/ka/syllable improved after the intervention. Furthermore, Iwao [15] showed a significant improvement in all syllables among older people aged 65–74 years.

Regarding oral exercises, Sugiyama [14] conducted tongue movement and utterance of some words in a clear yet slow manner, whereas Kito [18] only conducted tongue movement. Given that uttering three syllables is suitable for increasing the ODK rate per second, the ODK in the studies of Sugiyama [14] and Kito [18] without the exercise did not improve significantly.

Iwao [15] also conducted tongue movement and utterance of a multisyllable sound (/pataka/) similar to our study; however, the/ta/syllable did not significantly improve in people aged over 75 years. Although older people would be less likely to improve in oral function, this discrepancy between Iwao [15] and the present study is unclear. Further verification is necessary to determine the reason for this difference.

Given that the oral functions of the participants improved, their diet was also expected to improve. In particular, masticatory function improvement is advantageous for proper ingestion. However, the participants' diet did not significantly improve, indicating that oral function improvement does not always result in dietary improvement.

Although the frequency of ingesting green vegetable salad, green and yellow vegetables, cabbage/Chinese cabbage, and fruit increased, it did not reach statistical significance.

Our study data showed that enhanced masticatory function alone could not improve an individual's diet, similar to studies investigating dietary intake before and after providing prosthetics. Several intervention studies examined whether an intervention involving the provision of fitted dentures could improve food and/or nutrient intake in older adults; results showed no significant difference in the data before and after the intervention [24–26].

Conversely, some studies found that providing nutrition counseling in addition to prosthetic treatment increases fruit and vegetable intake in edentulous individuals [27–29]. Additionally, dietary counseling alone made the individuals' dietary habits change for 6 months [30]. The results of these studies and the present study suggest that oral function improvement alone would be insufficient in changing the diet of older people; moreover, the present study's period of intervention might be too short to change dietary habits. Intervention with dietary counseling as well as oral exercises would have a higher effect on older people's diet habit, and a longer intervention period would be necessary to change their diet.

Regarding the effect size, tongue pressure and ODK in this study were lower than those in previous studies [8, 9], possibly because the mean differences between pre- and postintervention were smaller.

Our participants might have low oral function. Although their oral function somehow improved after an oral exercise, an exercise to further strengthen the root of the tongue muscle was suggested to significantly improve the/ka/syllable.

This study is the first to investigate whether oral function improvement by oral function training could affect the diet of older adults. However, it has some limitations. First, it may have a selection bias because the participants were not selected randomly; they were recruited by district welfare commissioners, managers of welfare facilities, including day-care centers, and staff of the social welfare council. The older adults were considered to be health-conscious. The existence of a bias affecting the study results cannot be denied. The sampling method for selecting participants should be examined in the future. Second, although the method used for evaluating ingestion frequency was easy to utilize for older people and was commonly used for a large population survey, the quantitative data on dietary intake could not be obtained. In contrast, dietary recording or the 24 h recall method is useful for investigating dietary intake, but it bears substantial burden for them. Finally, the sample size was small. The number of participants was estimated by the effect size according to the methodology used in previous interventional studies on oral function. However, increasing the sample size is still necessary to clarify the effects of intervention on oral function and diet. Further research is also necessary to confirm whether oral function training with supplement treatment can improve the diet of older adults.

## 5. Conclusions

While oral function training improved tongue pressure/pa/and/ta/syllables, and masticatory force in 16 community-dwelling older people requiring support care levels 1 and 2, it did not significantly affect the dietary habits of these individuals. Therefore, in future studies with a larger sample size, oral function training should be combined with any dietary counseling to improve the dietary habits of older people.

## Figures and Tables

**Table 1:** Demographic factors *n* = 16.

Age (mean ± SD)	76.6 ± 8.04 (years)
Sex (male/female)	6/10
CSCL (1/2)	11/5

Abbreviation: CSCL, certified support care level.

**Table 2:** *p* value in Shapiro–Wilk test.

	Preintervention	Postintervention
Tongue pressure	0.705	0.425
ODK rate:Pa syllable	0.222	0.296
ODK rate:Ta syllable	0.886	0.447
ODK rate:Ka syllable	0.904	0.066
Masticatory force	0.254	0.848

**Table 3:** Oral function *n* = 16.

	**Unit**		**Mean ± SD**	**Mean difference ±SD**	**p**valu**e**^†^	**Effect size**

Tongue pressure	kPa	BL	29.3 ± 9.35	5.13 ± 4.04	< 0.001^∗∗^	0.547
		EP	34.4 ± 9.41			

ODK rate:Pa syllable	times/s	BL	5.2 ± 0.67	0.48 ± 0.78	0.004^∗^	0.589
		EP	5.6 ± 0.85			

ODK rate:Ta syllable	times/s	BL	5.2 ± 0.71	0.54 ± 0.99	0.018^∗^	0.699
		EP	5.7 ± 0.72			

ODK rate:Ka syllable	times/s	BL	5.1 ± 0.68	0.25 ± 0.54	0.894	0.353
		EP	5.3 ± 0.74			

Masticatory force	—	BL	27.5 ± 3.43	2.60 ± 3.58	0.012^∗^	0.803
		EP	30.1 ± 2.90			

^†^paired *t*-test.

^**∗**^ *p* < 0.05.

^**∗∗**^ *p* < 0.01.

**Table 4:** Frequency of food ingestion person (%) *n* = 16.

Foods/frequency		Zero times	Less than one per week	Once a week	2-3 times a week	4–6 times a week	Once a day	2 or more times daily	*p* value^∗^
Meals	BL	0 (0.00)	0 (0.00)	2 (0.13)	3 (0.19)	4 (0.25)	7 (0.44)	0 (0.00)	0.805
	EP	0 (0.00)	0 (0.00)	2 (0.13)	5 (0.31)	1 (0.06)	7 (0.44)	1 (0.06)	

Fish	BL	0 (0.00)	1 (0.06)	5 (0.31)	8 (0.50)	1 (0.06)	1 (0.06)	0 (0.00)	0.248
	EP	0 (0.00)	1 (0.06)	2 (0.13)	7 (0.44)	2 (0.13)	4 (0.25)	0 (0.00)	

Potato	BL	2 (0.13)	3 (0.19)	3 (0.19)	3 (0.19)	3 (0.19)	2 (0.13)	0 (0.00)	0.773
	EP	0 (0.00)	2 (0.13)	3 (0.19)	6 (0.38)	4 (0.25)	0 (0.00)	1 (0.06)	

Green vegetables salad	BL	0 (0.00)	1 (0.06)	2 (0.13)	3 (0.19)	2 (0.13)	5 (0.31)	3 (0.19)	0.204
	EP	0 (0.00)	0 (0.00)	1 (0.06)	4 (0.25)	1 (0.06)	7 (0.44)	3 (0.19)	

Green and yellow vegetable	BL	2 (0.13)	3 (0.19)	2 (0.13)	2 (0.13)	4 (0.25)	3 (0.19)	0 (0.00)	0.123
	EP	0 (0.00)	1 (0.06)	2 (0.13)	5 (0.31)	3 (0.19)	4 (0.25)	1 (0.06)	

Cabbage Chinese cabbage	BL	1 (0.06)	0 (0.00)	0 (0.00)	8 (0.50)	4 (0.25)	3 (0.19)	0 (0.00)	0.544
	EP	0 (0.00)	0 (0.00)	3 (0.19)	3 (0.19)	3 (0.19)	5 (0.31)	2 (0.13)	

Carrot pumpkin	BL	1 (0.06)	3 (0.19)	3 (0.19)	5 (0.31)	2 (0.13)	2 (0.13)	0 (0.00)	0.714
	EP	1 (0.06)	2 (0.13)	2 (0.13)	5 (0.31)	1 (0.06)	5 (0.31)	0 (0.00)	

Radish turnip	BL	0 (0.00)	2 (0.13)	4 (0.25)	10 (0.63)	0 (0.00)	0 (0.00)	0 (0.00)	0.773
	EP	0 (0.00)	2 (0.13)	5 (0.31)	6 (0.38)	2 (0.13)	1 (0.06)	0 (0.00)	

Other root crops	BL	1 (0.06)	0 (0.00)	3 (0.19)	8 (0.50)	2 (0.13)	3 (0.19)	0 (0.00)	0.564
	EP	0 (0.00)	3 (0.19)	1 (0.06)	4 (0.25)	2 (0.13)	5 (0.31)	1 (0.06)	

Seaweed	BL	1 (0.06)	1 (0.06)	3 (0.19)	9 (0.56)	2 (0.13)	0 (0.00)	0 (0.00)	0.472
	EP	0 (0.00)	4 (0.25)	3 (0.19)	5 (0.31)	1 (0.06)	2 (0.13)	1 (0.06)	

Fruit	BL	1 (0.06)	0 (0.00)	3 (0.19)	6 (0.38)	1 (0.06)	5 (0.31)	0 (0.00)	0.107
	EP	0 (0.00)	1 (0.06)	3 (0.19)	3 (0.19)	4 (0.25)	5 (0.31)	0 (0.00)	

Bread	BL	1 (0.06)	0 (0.00)	1 (0.06)	1 (0.06)	2 (0.13)	12 (0.75)	0 (0.00)	0.414
	EP	0 (0.00)	0 (0.00)	0 (0.00)	3 (0.19)	0 (0.00)	12 (0.75)	1 (0.06)	

*Note:* BL, baseline; EP, endpoint.

^∗^Wilcoxon signed-rank sum test.

## Data Availability

The research data are not shared, in accordance with the guidelines of the Ethics Committee of Hyogo University, which prohibit sharing the data with or offering them to any third parties.
